# Performing Magnetic Boundary Modulation to Broaden the Operational Wind Speed Range of a Piezoelectric Cantilever-Type Wind Energy Harvester

**DOI:** 10.3390/mi15111286

**Published:** 2024-10-23

**Authors:** Feng-Rui Liu, Lin-Chuan Zhao, Ge Yan, Wen-Ming Zhang, Zhi-Yuan Wu, Xiao-Long Zhang

**Affiliations:** 1 Shanghai Key Laboratory of Aerospace Intelligent Control Technology, Shanghai Aerospace Control Technology Institute, Shanghai 201109, China; 2 State Key Laboratory of Mechanical System and Vibration, School of Mechanical Engineering, Shanghai Jiao Tong University, Shanghai 200240, China

**Keywords:** energy harvesting, wind, stepped beam, magnetic boundary, modulation

## Abstract

Small piezoelectric wind-induced vibration energy harvesting systems have been widely studied to provide long-term sustainable green energy for a large number of wireless sensor network nodes. Piezoelectric materials are commonly utilized as transducers because of their ability to produce high output power density and their simple structure, but they are prone to material fracture under large deformation conditions. This paper proposes a magnetic boundary modulated stepped beam wind energy harvesting system. On the one hand, the design incorporates a composite stepped beam with both high- and low-stiffness components, allowing for efficient vibration and electrical energy output at low wind speeds. On the other hand, a magnetic boundary constraint mechanism is constructed to prevent the piezoelectric sheet from breaking due to excessive deformation. Experiments have confirmed that the effective operational wind speed range of the harvester with magnetic boundary constraints is doubled compared to that of the harvester without magnetic boundary constraints. Furthermore, by adjusting the magnetic pole spacing of the boundary, the harvesting system can generate sufficiently high output power under high-wind-speed conditions without damaging the piezoelectric sheet.

## 1. Introduction

With the rapid development of the Internet of Things technology, a large number of micro sensors have been used in many human-related occasions such as agricultural monitoring, environmental protection, industrial manufacturing, and human health monitoring [1,2,3]. Most of these sensors are powered by chemical batteries to avoid the trouble caused by long-distance wiring [4,5,6]. However, the capacity of chemical batteries is very limited and needs to be replaced frequently. In certain harsh environments, the cost of manual maintenance is very high, and it is easy to cause environmental pollution. Some small energy harvesting systems use widely distributed green energy, such as wind energy, solar energy, and mechanical energy, to provide long-term sustainable electricity for sensors [7,8,9,10,11,12,13,14]. Among them, the wind-induced vibration energy harvesting system utilizes wind-induced vibration mechanisms such as flutter [15], vortex-induced vibration (VIV) [16,17,18,19], galloping [20,21,22], and wake galloping [23] to convert the structural vibration energy caused by wind into electrical energy. Because the system is relatively simple, it has become an important research and development direction [24,25].

Electromagnetic [26], triboelectric [27,28,29,30], and piezoelectric [31,32,33] energy conversion methods are often used to convert the vibration energy of the wind-induced vibration energy harvester into electrical energy. The electromagnetic energy harvesting system uses the relative movement between the coil and the magnet to generate electromagnetic induction for energy conversion. The energy conversion sheet often features a complex structure, substantial size, difficulty in miniaturization, and low induced voltage. The triboelectric energy harvesting system is a system that allows two materials with different electron gain and loss capabilities to contact or rub against each other, resulting in electron transfer and generating a current in an external load circuit. This mechanism has a higher output voltage but a lower output current. Piezoelectric energy harvesting utilizes the piezoelectric effect of some materials, such as lead zirconate titanate piezoelectric ceramics, polyvinylidene fluoride, zinc oxide, and other piezoelectric materials. When external forces are applied to these materials, their polarization changes, leading to the formation of positive and negative charges on their surfaces. This mechanism can easily output a high voltage under weak excitation conditions and has a high output power density per unit volume.

For achieving high power density and efficiency in wind energy harvesting, piezoelectric transduction has emerged as one of the most attractive energy harvesting mechanisms [24,34,35,36]. However, excessive deformation of the cantilever beam will cause microcracks in the piezoelectric sheet, reducing its energy harvesting performance [37,38,39]. To ensure the long-term operational durability of the piezoelectric sheet, the vibrational deformation of the cantilever beam must be restricted within a safe range. Additionally, homogeneous cantilever beam structures, due to their simplicity, are widely used in traditional piezoelectric wind energy harvesters. Wind energy harvesters with low bending stiffness in the cantilever beam can achieve a low cut-in wind speed, but their maximum allowable operating wind speed is relatively low, as high wind speeds can cause excessive deformation of the piezoelectric sheets attached to the beam, increasing the risk of structural failure. On the other hand, harvesters with high bending stiffness can operate stably at higher wind speeds, but their cut-in wind speed is too high to be effective in low-wind conditions.

The introduction of magnetic forces can enhance the dynamic response characteristics of wind-induced vibration energy harvesting systems. For example, Naseer et al. [16] developed a lumped parameter model for a VIV (vortex-induced vibration) wind energy harvester, where they theoretically introduced nonlinear magnetic attraction to modify the lock-in region of the VIV-based energy harvester. Nonlinear dynamic analysis revealed that adjusting the distance between two magnets improved the system’s output performance. Zhang et al. [40] attached two magnets with a repulsive force, respectively, on a lower support and the bottom of a circular cylinder. Experiments show that the proposed energy harvester displays a softening behavior due to the impact of nonlinear magnetic forces, which shifts the synchronization region and increases the harvesting performance. Zhao et al. [41] proposed a two-degree-of-freedom (2DOF) piezoelectric aeroelastic energy harvester with a cut-out cantilever and two magnets. Translational galloping is induced with a square cross-sectioned bluff body attached at the cantilever tip. Magnetic interaction is introduced to create stiffness nonlinearity, which makes the harvester have a lower cut-in wind speed of 1 m/s. Zhou et al. [18] enhanced the VIV-based wind energy harvesting system by leveraging the tristable characteristics induced by magnetic forces. Both the theoretical and experimental results show that the wind speed range of the system was expanded by more than double, and the harvesting voltage increased by 30%. Qin et al. [42] added a tip magnet and two fixed magnets to introduce multi-stability to the wind harvesting device, which includes two square cylinders and a circular cylinder. By introducing multi-stability and combining the advantages of VIV and galloping, the system could maintain a large amplitude vibration over a wide range of wind speed and generate a high electric output. Magnetic forces are generally applied to the free end of the energy harvester to change the stiffness or steady-state characteristics of the system. In this manuscript, magnetic forces are introduced as a form of nonlinear boundary constraint to improve dynamic responses and prevent fracture in the piezoelectric sheet. A stepped cantilever beam design is adopted to achieve cut-in wind speeds at low levels, broadening the operational wind speed range of the harvesting system.

## 2. Conceptual Design

To improve the operational wind speed range, a magnetic boundary-modulated stepped beam is proposed for the wind energy harvester. Unlike the aforementioned homogeneous beam, the stepped beam consists of two sections with different stiffnesses: one half is a high-stiffness beam, and the other half is a low-stiffness beam. The low-stiffness section allows the harvester to vibrate and produce stable energy output at low wind speeds. When the stepped beam vibrates with a large amplitude, the repulsive force generated by the magnetic boundary restricts the deformation of the piezoelectric sheet within a safe range, effectively doubling the operating wind speed range.

**Figure 1:**
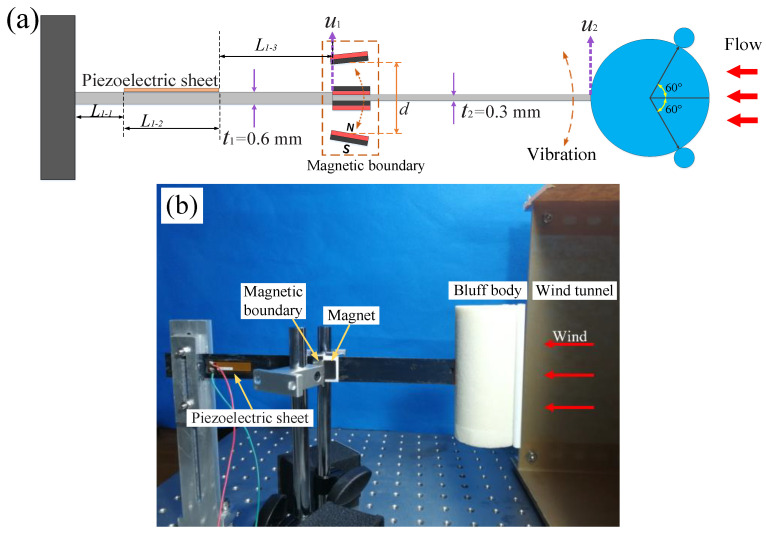
(**a**) A schematic diagram of the wind energy harvester with a magnetic boundary modulated stepped beam; (**b**) the experimental setup.

As shown in Figure 1a, the stepped cantilever beam of the harvester consists of two 65Mn spring steel beams with different thicknesses ($t_{1}=0.6\;mm\;and\;t_{2}=0.3\;mm$). They have the same heat treatment process and material strength. The two cantilever sections have the same length and width, with a bending stiffness ratio of $EI_{1}/EI_{2}=t_{1}^{3}/t_{2}^{3}=8$ between them. The high-stiffness beam is fixed at the base of the harvester, while the low-stiffness beam is attached to a bluff body at the free end. They are bonded together with a high-strength AB epoxy adhesive. Two permanent magnets are fixed on either side of the connection point between the high- and low-stiffness beams. Additionally, two other magnets, which serve as magnetic boundary constraints, are symmetrically positioned at an equal distance (d/2) from both sides of the cantilever beam. The magnetic force between the cantilever beam and the boundary magnets generates a repulsive force.

In the experiment, the bluff body of the harvester is cylindrical with two small cylindrical rods (5 mm in diameter) attached to the windward side, a structure that enhances the aerodynamic response range of vortex-induced vibration (VIV) [43]. The length and diameter of the main cylinder are 10 cm and 5 cm, respectively. A piezoelectric sheet (Smart Material Corp., MFC-M2807-P2, Sarasota, FL, USA) is bonded near the base of the cantilever beam, and an external resistor of R = ${1\times 10}^{6}\;\Omega$ is connected. The performance of the wind energy harvester was tested experimentally in an open wind tunnel with a cross-sectional area of 35 cm × 30 cm, and data were recorded using the Donghua data acquisition system (Donghua Testing Technology Corp., DH5902, Jingjiang, China).

Since the repulsive force between the magnets decreases sharply with an increasing distance, the magnetic repulsion acting on the cantilever beam is weak when the vibration amplitude is small. However, when the amplitude is larger, the magnetic boundary exerts a relatively stronger repulsive force on the cantilever beam, thereby confining its amplitude within the boundary. Consequently, at higher wind speeds, the magnetic force can limit the vibration amplitude of the high-stiffness section ($t_{1}=0.6\;mm$) connected to the piezoelectric sheet, preventing excessive deformation and avoiding fracture in the piezoelectric sheet. The displacement at the free end of the high-stiffness section of the cantilever beam is denoted as $u_{1}(t)$, while the displacement at the free end of the low-stiffness section ($t_{2}=0.3\;mm$) is denoted as $u_{2}(t)$. The length of the cantilever stepped beam is L. It can be divided into high-stiffness and low-stiffness parts with different cross sections and lengths of $L_{1}$, $L_{2}$($L_{1}+L_{2}=L$). The piezoelectric sheet is bonded on the high-stiffness part. The high-stiffness part can be divided into three parts with different cross sections and lengths of $L_{1-1}$, $L_{1-2}$, $L_{1-3}$($L_{1-1}+L_{1-2}+L_{1-3}=L_{1}$) (Figure 1a).

Based on the Euler–Bernoulli beam theory, the bending vibration equation for the stepped beam can be established as follows:

(1) $$\begin{array}{c} E_{i}I_{i}\frac{\partial^{4}u_{i}(x,t)}{\partial x^{4}}+m_{i}\frac{\partial^{2}u_{i}(x,t)}{\partial t^{2}}+(\frac{d\delta (x-L_{1-1})}{dx}-\frac{d\delta (x-L_{1-2})}{dx})\vartheta_{p}V(t)\;\;\\ =F_{m}\delta (x-L_{1})+F_{air}\delta (x-L) \end{array}$$

$E_{i}$ is the elastic modulus of each part (i=1, 2, 1−1, 1−2, 1−3), $I_{i}=b_{i}h_{i}^{3}/12$ is the cross-sectional inertia, $m_{i}$ is the mass per unit length, $F_{m}$ is the magnetic force acting on the cantilever beam, and $F_{air}$ is the air force on the cantilever beam. The galloping-based wind energy harvesting system mainly excites the first-order mode of the cantilever beam structure, which is mainly analyzed in this paper. The first-order mode function of each part can be expressed as

(2) $$\begin{array}{c} \phi_{i}(x)=A_{i}\sin(\beta_{i}\cdot x)+B_{i}\cos(\beta_{i}\cdot x)\;\;\\ +C_{i}\sinh(\beta_{i}\cdot x)+D_{i}\cosh(\beta_{i}\cdot x) \end{array}$$

The displacement of any point on the cantilever beam can be expressed as

(3) $$u_{i}(x,t)=\phi_{i}(x)\cdot Q(t)$$

ω is the angular frequency of the cantilever beam. The mechanical continuity conditions between different parts imply the continuity of displacement, rotations, shear forces, and bending moment.

(4) $$\begin{array}{l} x=0:\\ \;\;\;\;\;\phi_{1-1}(0)=0,\;{\phi_{1-1}}^{\prime }(0)=0\\ x=L_{1-1}:\\ \;\;\;\;\;\phi_{1-1}(L_{1-1})=\phi_{1-2}(0),\;{\phi_{1-1}}^{\prime }(L_{1-1})={\phi_{1-2}}^{\prime }(0),\;\\ \;\;\;\;\;EI_{1-1}{\phi_{1-1}}^{\prime \prime }(L_{1-1})=EI_{1-2}{\phi_{1-2}}^{\prime \prime }(0),\;\\ \;\;\;\;\;EI_{1-1}{\phi_{1-1}}^{\prime \prime \prime }(L_{1-1})=EI_{1-2}{\phi_{1-2}}^{\prime \prime \prime }(0)\\ x=L_{1-1}+L_{1-2}:\;\\ \;\;\;\;\;\phi_{1-2}(L_{1-1}+L_{1-2})=\phi_{1-3}(0),\;{\phi_{1-2}}^{\prime }(L_{1-1}+L_{1-2})={\phi_{1-3}}^{\prime }(0),\;\\ \;\;\;\;\;EI_{1-2}{\phi_{1-2}}^{\prime \prime }(L_{1-1}+L_{1-2})=EI_{1-3}{\phi_{1-3}}^{\prime \prime }(0),\;\\ \;\;\;\;\;EI_{1-2}{\phi_{1-2}}^{\prime \prime \prime }(L_{1-1}+L_{1-2})=EI_{1-3}{\phi_{1-3}}^{\prime \prime \prime }(0)\\ x=L_{1}:\\ \;\;\;\;\;\phi_{1}(L_{1})=\phi_{2}(0),\;{\phi_{1}}^{\prime }(L_{1})={\phi_{2}}^{\prime }(0),\;\\ \;\;\;\;\;EI_{1}{\phi_{1}}^{\prime \prime }(L_{1})=EI_{2}{\phi_{2}}^{\prime \prime }(0),\;\\ \;\;\;\;\;EI_{1}{\phi_{1}}^{\prime \prime \prime }(L_{1})=EI_{2}{\phi_{2}}^{\prime \prime \prime }(0)-\omega^{2}M_{m}\phi_{2}(0)\\ x=L:\\ \;\;\;\;\;EI_{2}{\phi_{2}}^{\prime \prime \prime }(L)+\omega^{2}M_{t}\phi_{2}(L)=0\\ \;\;\;\;\;EI_{2}{\phi_{2}}^{\prime \prime }(L)-\omega^{2}I_{t}{\phi^{\prime }}_{2}(L)=0 \end{array}$$

The normalization condition of the mode function is

(5) $$\begin{array}{c} \int_{0}^{L_{1-1}}{\phi_{1-1}}^{2}(x)m_{1-1}dx+\int_{L_{1-1}}^{L_{1-1}+L_{1-2}}{\phi_{1-2}}^{2}(x)m_{1-2}dx+\int_{L_{1-1}+L_{1-2}}^{L_{1}}{\phi_{1-3}}^{2}(x)m_{1-3}dx+\;\;\\ {\phi_{2}}^{2}(0)M_{m}+\int_{L_{1}}^{L_{2}}{\phi_{2}}^{2}(x)m_{2}dx+{\phi_{2}}^{2}(L)M_{t}+{{\phi^{\prime }}_{2}}^{2}(L)I_{t}=1 \end{array}$$

According to Equations (2), (4) and (5), the values of the coefficients $A_{i},\;B_{i},\;C_{i},\;D_{i}$ can be determined, and the first-order mode function $\phi_{i}(x)$ of each part can be obtained using Matlab 2018. And then, the first-order frequency ω can be calculated by

(6) $$\begin{array}{c} \int_{0}^{L_{1-1}}{\left(\frac{d^{2}\phi_{1-1}(x)}{dx^{2}}\right)}^{2}EI_{1-1}dx+\int_{L_{1-1}}^{L_{1-1}+L_{1-2}}{\left(\frac{d^{2}\phi_{1-2}(x)}{dx^{2}}\right)}^{2}EI_{1-2}dx+\;\;\;\;\\ \int_{L_{1-1}+L_{1-2}}^{L_{1}}{\left(\frac{d^{2}\phi_{1-3}(x)}{dx^{2}}\right)}^{2}EI_{1-3}dx+\int_{L_{1}}^{L}{\left(\frac{d^{2}\phi_{2}(x)}{dx^{2}}\right)}^{2}EI_{2}dx=\omega^{2} \end{array}$$

By substituting Equation (3) and $\phi_{i}(x)$ into Equation (1), the normalized electromechanical coupling dynamic model can be obtained as follows:

(7) $$\begin{array}{l} \ddot{Q}(t)+2\xi \omega \dot{Q}(t)+\omega^{2}Q(t)+\theta_{p}V(t)=f_{air}(t)+f_{m}(t)\\ C_{p}\dot{V}(t)+\frac{V(t)}{R}-\theta_{p}\dot{Q}(t)=0 \end{array}$$

where ξ is the mechanical damping coefficient, which is calculated through the free vibration test of a cantilever beam. $\theta_{p}=(\phi^{\prime }(L_{1-2})-\phi^{\prime }(L_{1-1}))\vartheta_{p}$ is the electromechanical coupling coefficient, $C_{p}$ is the capacitance of the piezoelectric sheet, and R is the external resistance.

The normalized air force $f_{air}(t)$ is expressed as

(8) $$f_{air}(t)=\phi_{2}(L)F_{air}$$

where $F_{air}$ is the air force acting on the bluff body. The normalized magnetic force $f_{mag}(t)$ is expressed as

(9) $$f_{m}(t)=\phi_{2}(0)F_{m}$$

where $F_{m}$ is the magnetic force acting on the cantilever beam.

## 3. Experimental Comparison

**Figure 2:**
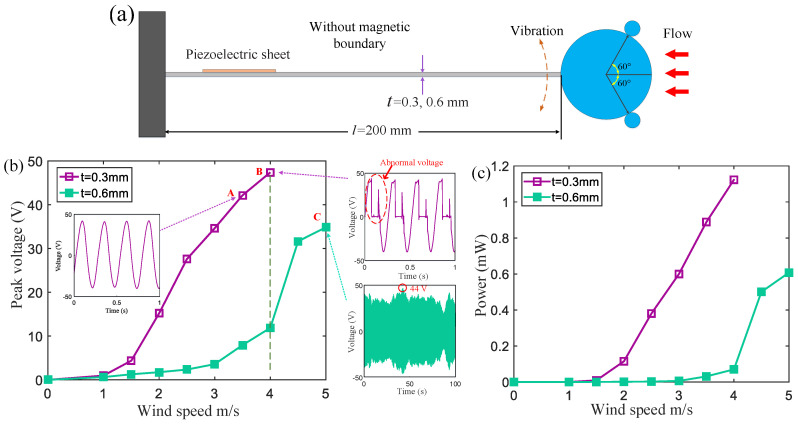
(**a**) A schematic diagram of a piezoelectric wind energy harvester with a homogeneous cantilever beam (the thicknesses of the cantilever beams are 0.3 mm and 0.6 mm, respectively); (**b**) the average peak voltages and (**c**) power of the two corresponding wind energy harvesters.

For comparison, the output voltages of wind energy harvesters with high-stiffness and low-stiffness homogeneous cantilever beams were first measured (see Figure 2). The sizes of the two cantilever beams are 200 mm × 20 mm × 0.3 mm (low-stiffness beam) and 200 mm × 20 mm × 0.6 mm (high-stiffness beam).

Figure 2b,c show the average peak voltage and power generated by two wind energy harvesters with different stiffness beams. Based on a low-stiffness cantilever beam with a thickness of t=0.3 mm, the wind energy harvester can generate a high output voltage at a low wind speed (purple curve). At a wind speed of 2 m/s, its output voltage is 15 V, and at a wind speed of 3.5 m/s, the output voltage reaches about 42 V (point A in Figure 2b). When the wind speed further increases to 4 m/s, the peak voltage reaches about 47 V (point B in Figure 2b). However, after about five minutes, the piezoelectric sheet is too deformed to cause structural cracks and can no longer output a normal continuous voltage (the right inset of the voltage at point B in Figure 2b). The experiment shows that excessive tensile deformation leads to piezoelectric materials with a very short cycle life.

A homogeneous high-stiffness cantilever beam (t=0.6 mm) is employed to harvest wind energy. When the wind speed increases to 4 m/s, the energy harvester can still output electrical energy normally (green curve), but the average peak voltage of 12 V is much lower than that of the harvester based on a low-stiffness cantilever beam. When the wind speed is 5 m/s, the average peak voltage reaches about 35 V, and the peak voltage fluctuates over time (see the inset depicting the voltage at point C in Figure 2b). The maximum voltage is approximately 44 V. If the wind speed further increases, the piezoelectric sheet may sustain damage due to excessive deformation. The experimental results demonstrate that wind energy harvesters utilizing high-rigidity beams can only operate within the range of 0 to 5 m/s.

**Figure 3:**
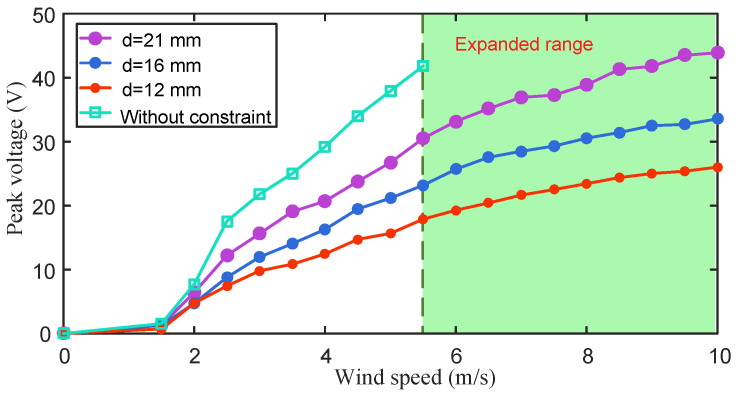
The output voltage of the wind energy harvester with three different magnetic boundary distances in the wind speed range of 0~10 m/s. The green curve refers to the peak voltage of the stepped beam without the magnets attached.

Subsequently, the energy harvesting performance of the stepped cantilever beam without the magnets attached was tested (green curve in Figure 3). The dimensions of the two parts of the stepped beam are 100 mm × 20 mm × 0.3 mm (low-stiffness part) and 100 mm × 20 mm × 0.6 mm (high-stiffness part) (see Figure 1a). The results show that compared with the harvester using a homogeneous high-stiffness beam, the harvester with the stepped cantilever beam has a lower starting wind speed, and the average peak voltage can reach about 14 V at 2.2 m/s. However, when the wind speed is 5.5 m/s, the average peak voltage can reach 40 V (Figure 3). If the wind speed continues to increase, the piezoelectric sheet may be damaged due to excessive deformation.

In order to further expand the wind speed range, magnetic boundary constraints are introduced on both sides of the wind energy harvester based on the stepped beam (see Figure 1). The dimensions of the two magnets on the beam are 20 mm × 10 mm × 1 mm, and the dimensions of the two magnets on the boundary are 20 mm × 10 mm × 2 mm. The output performance of the wind energy harvester with magnetic boundary constraints was measured in the range of 0~10 m/s, as shown in Figure 3. The distances between the two magnetic boundaries were, respectively, d=12,16,21 mm, and the wind energy harvester performed well under the three magnet distance conditions. The effective wind speed range of the energy harvester was expanded to 10 m/s, which is twice that of the case without magnetic boundary constraints. The larger the magnet spacing, the higher the harvester output voltage. As shown in Table 1, when the boundary distance is adjusted to d=21 mm, in the range of 0 to 5 m/s, the average peak voltage reaches more than 70% of the stepped beam without magnetic boundaries.

**Figure 4:**
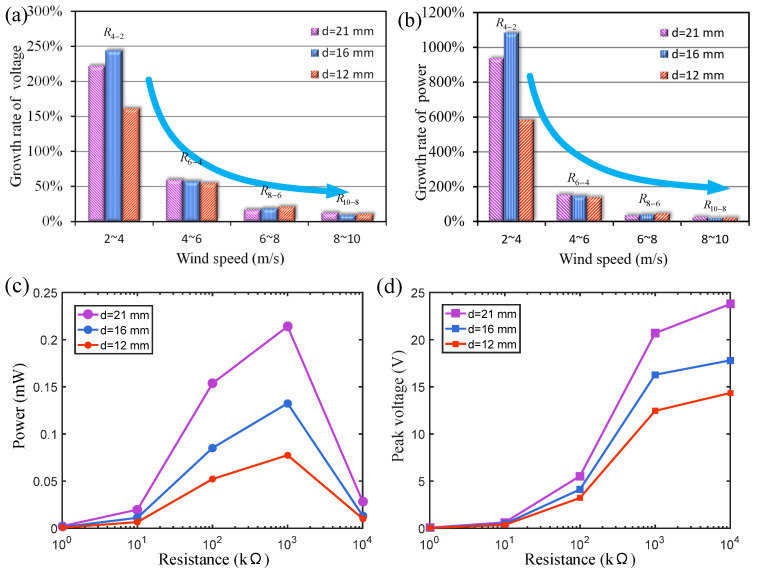
(**a**) The output voltage and (**b**) power growth rate of the energy harvester under three different magnet distances (magnet thickness of 1 mm); (**c**,**d**) the influence of external resistance on the output peak voltage and power at a 4 m/s wind speed.

The output voltage growth rates of wind energy harvesters in different wind speed ranges are compared, as shown in Figure 4a. The growth rate is expressed as $R_{j-i}=(V_{U=j}-V_{U=i})/V_{U=i}$, where $V_{U=j}$ and $V_{U=i}$ represent the output voltages at the initial wind speed (U=i) and the final wind speed (U=j) within a certain wind speed range, respectively. In the wind speed range of 2~4 m/s, the voltage growth rate $R_{4-2}$ of wind energy harvesters with three different magnetic distances exceeds 150%. In Figure 4b, the power growth rate is expressed as $R_{P,j-i}=(P_{U=j}-P_{U=i})/P_{U=i}$, where $P_{U=j}$ and $P_{U=i}$ represent the output voltages at the initial wind speed (U=i) and the final wind speed (U=j) within a certain wind speed range, respectively. Under such wind speed conditions, the vibration amplitude of the cantilever beam is small, and the magnetic repulsion it receives is weak. The output voltage of the harvester can rise steadily with the increase in wind speed. As the vibration amplitude increases, the magnetic repulsion generated by the magnetic boundary on the cantilever increases significantly, and the voltage growth rate decreases accordingly. When the wind speed increases from 8 m/s to 10 m/s, under the strong magnetic boundary constraint, the vibration amplitude of the harvester no longer increases significantly, and the voltage growth rate is only 7%. The voltage growth rate under a high wind speed is very small. The results show that the magnetic boundary can effectively constrain the vibration amplitude of the cantilever beam. Because the magnetic force is not a rigid hard contact, this constraint will not destroy the vibration state of the cantilever beam.

For comparison, we also tested a mechanical hard boundary constraint scheme without magnets to limit the vibration amplitude of the harvester. The experimental results show that the collision between the wind energy harvester and the rigid hard boundary will destroy the stability of the cantilever beam vibration, causing the energy harvesting output to fluctuate very much. The nonlinear force generated by the magnetic confinement boundary can maintain the stable vibration of the cantilever beam and avoid violent fluctuations in the output energy.

Figure 4c,d show the influence of external resistance on the output peak voltage and power at a 4 m/s wind speed under three different magnetic pole spacings (d=21, 16 and 12 mm). The results indicate that when the external resistance load is around 1 MΩ, the energy harvester obtains the best output power at 4 m/s. The optimal resistance for maximizing the output power of the wind energy harvester may vary with the wind speed. In this paper, the resistance is fixed at 1 MΩ to focus on comparing the effects of the cantilever beam type (homogeneous beam or stepped beam) and magnetic spacing on the output performance of the wind energy harvester.

## 4. Dynamics Analysis of Magnetic Boundary Modulation

**Figure 5:**
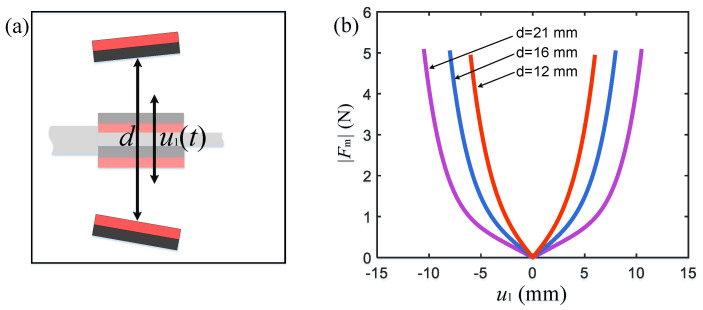
Relationship between magnetic repulsion and distance under different magnetic pole spacing conditions. (**a**) Magnetic pole spacing description; (**b**) magnetic force curves with different magnetic pole spacings.

The mathematical relationship between the magnetic boundary constraint force and the displacement $u_{1}$ of the cantilever beam can be approximated by a high-order polynomial as follows [44]:

(10) $$F_{m}(u_{1})=\sum_{i=0}^{n}a_{i}[{\left(d/2+u_{1}\right)}^{i}-{\left(d/2-u_{1}\right)}^{i}],$$

where $F_{m}$ is the magnetic force acting on the cantilever beam from the magnetic boundaries, $u_{1}$ is the displacement at the position where the magnet is attached to the cantilever beam, and d is the magnetic pole spacing. $(d/2+u_{1})$ and $\left(d/2-u_{1}\right)$ are the relative distances between two magnetic boundaries and the cantilever beam of the energy harvester, respectively (Figure 5a). n is the order of the polynomial equation. In order to accurately represent the magnitude of the magnetic force, n=9 is taken here. $a_{0}$–$a_{n}$ are the fitting coefficients of the fitting polynomial, which need to be determined by fitting based on measurement data. After measuring the nonlinear magnetic force at different displacements using a force transducer, the relationship between the magnetic boundary constraint force and the displacement was fitted by Equation (10) as shown in Figure 5b, and the fitting coefficients are shown in Table 2.

Based on the quasi-steady-state assumption, the air lift force $F_{\mathrm{air}}$ on the bluff body is expressed as [45]

(11) $$F_{\mathrm{air}}=\frac{1}{2}\rho U^{2}d_{b}l_{b}[a_{1}\frac{{\dot{u}}_{2}}{U}-a_{3}{\left(\frac{{\dot{u}}_{2}}{U}\right)}^{3}],$$

where $\rho =1.0{\;g/\mathrm{cm}}^{3}$ and U are the air density and wind speed, respectively. $d_{b}=(0.05+0.005)\cdot (\sqrt{3}/2)+0.005=0.0526\;m$ and $l_{b}=0.1\;m$ are the cross-sectional diameter and height of the bluff body, respectively. The aerodynamic coefficients $a_{1}$ and $a_{3}$ are set to 2.3 and 18.

The density of the stepped beam is $7.8\times 10^{3}{\;\mathrm{kg}/m}^{3}$, the elastic modulus of the beam is $2.1\times 10^{11}\;\mathrm{Pa}$, the electromechanical coupling coefficient is $\theta =3.0\times 10^{-4}\;N/V$, and the capacitance of the piezoelectric sheet is 15.1 nF. Based on the free vibration test, the equivalent damping ratios of the two beams with different stiffness values are measured to be $\zeta_{eq1}=0.001$ and $\zeta_{eq2}=0.008$, respectively.

**Figure 6:**
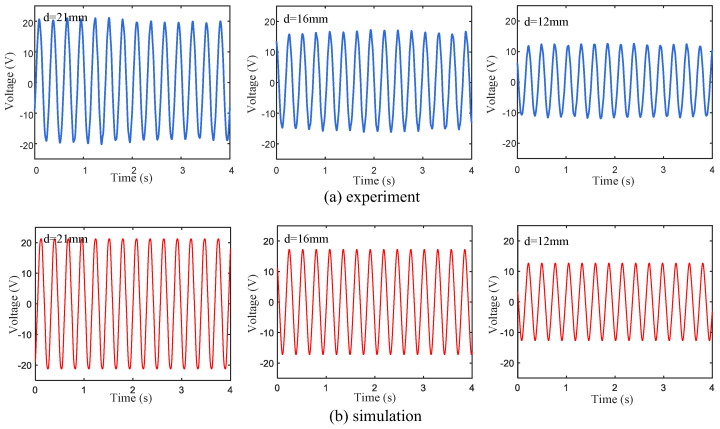
(**a**) Experimental and (**b**) simulated voltage responses of wind energy harvester based on three different magnetic pole spacings.

Based on the above electromechanical coupling Equations (1)–(11), the output voltages of the cases with different magnetic distances (d=12, 16 and 21 mm) under 4 m/s wind speed conditions were simulated by Matlab, and the results were compared with the experimental data (see Figure 6). Although the peak voltage obtained in the experiment fluctuates slightly, the voltage curves under different magnetic distance conditions are basically consistent with the simulation results.

The vibration characteristics of wind energy harvesters based on low-stiffness homogeneous beams (t=0.3 mm) and stepped beams under magnetic boundary constraints were compared and analyzed experimentally. This study found that the magnetic boundary can make the operational wind speed range of the harvester based on the homogeneous beam close to 5.5 m/s. However, when the wind speed increases further, the vibration amplitude of the cantilever beam continues to increase, and the piezoelectric sheet breaks.

**Figure 7:**
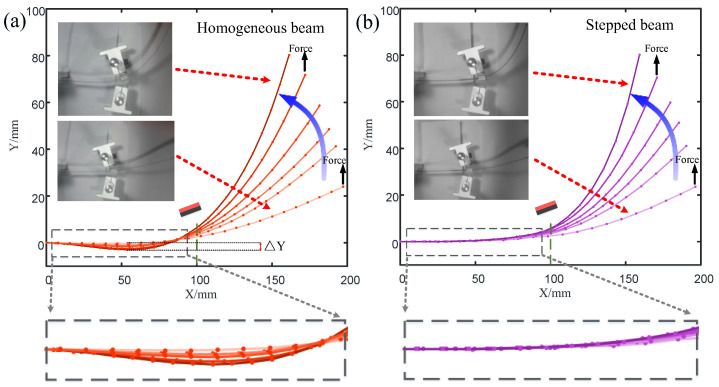
Deformations of a magnetically constrained homogeneous beam (t=0.3 mm) and a stepped beam when subjected to different lateral displacements. (**a**) A homogeneous beam (t=0.3 mm); (**b**) a stepped beam.

In order to explain the experimental results in Figure 6, the deformations of the homogeneous beam and the step beam under lateral external load were also tested (Figure 7). The transverse forces were applied to the free ends of the homogeneous beam and the step beam, respectively, to cause the cantilever beam to bend transversely from the equilibrium position. The force gradually increased until the transverse displacement of the free end of the cantilever beam reached 25, 40, 50, 60, 70, and 80 mm, respectively. The deformation processes of the magnetically constrained homogeneous beam (t=0.3 mm) and stepped beam were recorded and analyzed using cameras and image processing techniques. The distance of the magnetic boundary is d=16 mm. When the displacement of the end of the cantilever beam was relatively small (25 mm), the deformation difference between the homogeneous beam and the stepped beam was not obvious. As the end displacement increased, the homogeneous beam part in the dotted box in Figure 7a was concave in the opposite direction. However, for the part at the same position on the stepped beam in the dashed box (see Figure 7b), the corresponding deformation was very small. Obviously, when the displacement of the end of the beam was 80 mm, for the homogeneous beam, the negative lateral displacement at 54 mm from the root of the beam reached ΔY=−3 mm. The lateral displacement (ΔY=0.7 mm) at the same position on the stepped beam was small and positive. This shows that within a certain range, the magnetic boundary can effectively constrain the deformation of the stepped beam, while the low-stiffness beam will still undergo significant deformation.

**Figure 8:**
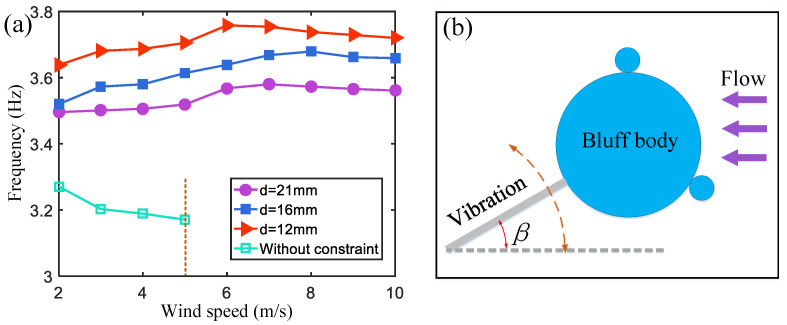
(**a**) The frequency of the wind energy harvester without a magnetic boundary and with three different magnetic spacings. (**b**) A schematic diagram of the cantilever beam tip rotated by an angle.

As shown in Figure 8, as the wind speed increases, the vibration frequency of the harvester without magnetic boundary constraints decreases, affecting the output power. After applying magnetic boundary constraints, the existence of magnetic repulsion on the cantilever beam increases the stiffness of the cantilever beam and the corresponding frequency ($f=\sqrt{k/m}$). Since the stiffness change is the largest, the magnetic boundary with a magnetic pole spacing of d=12 mm produces the largest repulsion, and the vibration frequency of the wind energy harvester is also the highest (purple curve in Figure 8a). The vibration frequency of the cantilever beam increases with the increase in wind speed. This is because the larger the vibration amplitude, the greater the magnetic repulsion force on the cantilever beam, which shortens the time it takes for the cantilever beam to return to its initial position.

When the wind speed increases further, the increase in the rotation angle β of the cantilever end will lead to a stronger air damping force. It extends the time for the cantilever to rebound to the initial position, causing the vibration frequency to slightly decrease when the wind speed is in the range of 8~10 m/s. However, at this time, the vibration frequency of the harvester remains higher than that observed at a wind speed of 2 m/s. The increase in the vibration frequency is conducive to improving the output power of the wind energy harvester.

## 5. Magnetic Boundary Modulation Verification

**Figure 9:**
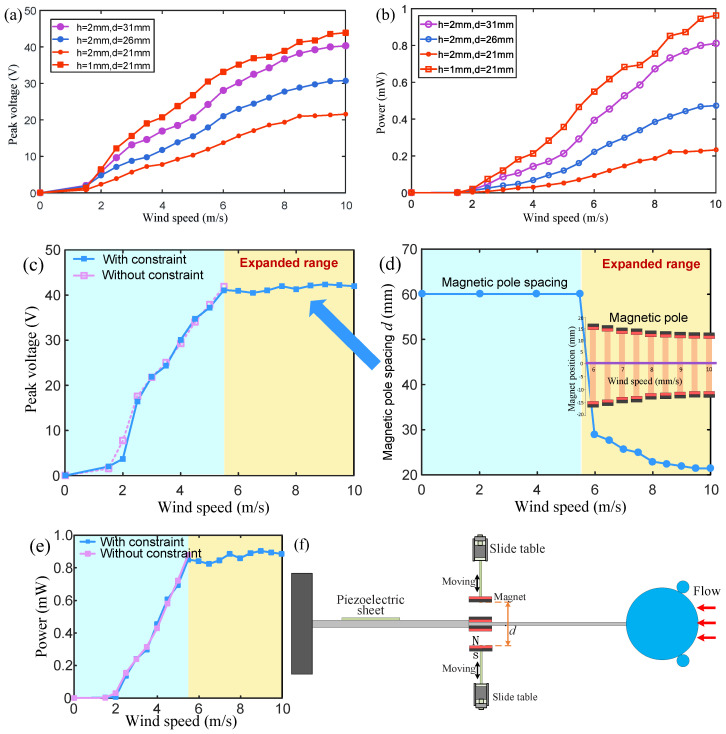
(**a**) The output voltage and (**b**) power of wind energy harvesters with different magnet thicknesses. (**c**) The output voltage and (**e**) power of the wind energy harvesters. The blue and purple curves represent the wind speed–voltage relationship with and without magnetic boundary constraints, respectively. (**d**) The adjusted magnetic pole spacing at different wind speeds. (**f**) The positions of the two magnets at the magnetic constraint boundary are adjusted by means of the electric sliding rails.

In order to further study the effect of the magnetic field intensity on the wind energy harvester, the two magnets (20 mm in length, 10 mm in width, and a thickness of h = 1 mm) fixed on the stepped beam were replaced with two larger magnets (with a thickness of h = 2 mm) with the same magnetization intensity, and the output performance of the wind energy harvester was then measured in the range of 0~10 m/s (see Figure 9a,b).

When the magnetic pole spacing is d=21 mm, the thick magnet causes the output voltage to drop to about 50% of the voltage generated by using thin magnets. When the magnetic pole spacing increases to d=26 mm, the output voltage increases to about 60% of the original voltage. When the spacing is further increased to d=31 mm, the output voltage can increase to about 90% of the original voltage in the wind speed range of 7~10 m/s. In the wind speed range of 7~10 m/s, the output power reaches about 80% of the original power (Figure 9b), which means that when the magnetic field is strong, a higher output voltage can also be obtained by increasing the boundary distance.

In addition, another test was conducted to demonstrate the advantages of the harvester with the magnetic boundary modulation stepped beam (Figure 9c–f). In this experiment, the wind speed gradually increased from 0 m/s to 10 m/s. After the wind speed exceeded 5.5 m/s, the output voltage could be maintained between 40 and 42 V as the wind speed increased by gradually reducing the magnetic pole spacing from 29 mm at 6 m/s to 21.5 mm at 10 m/s (the blue curve in Figure 9c). During the increase in wind speed from 0 m/s to 5.5 m/s, the magnetic pole spacing was fixed at 60 mm, which was sufficiently large to ensure that the magnetic repulsion force on the cantilever beam was nearly zero. In the experiment, the positions of the two magnets at the boundary of the magnetic force constraint were adjusted using the electric sliding rails (Figure 9f). Experiments have shown that the proper adjustment of the magnetic pole spacing can not only avoid the breakage of the piezoelectric sheet, but also make the harvester output efficiency as high as possible. This adjustment does not require changing the main structure and materials of the harvester and has strong adaptability and flexibility in different wind speed environments.

## 6. Conclusions

In this paper, a wind energy harvester with a stepped beam modulated by a magnetic boundary was designed and verified. The magnetic confinement boundary can not only prevent the piezoelectric sheet from breaking due to excessive deformation, but also maintain the stability of the output power in a wider wind speed range. Therefore, the harvesting system can relax the high-fracture-resistance requirements of the piezoelectric sheet. The low-stiffness part of the stepped beam helps the wind energy harvester vibrate and generate a high output voltage at low wind speeds. The high-stiffness part can limit the deformation of the piezoelectric sheet to a safe range. The effective wind speed range of the harvester using this structure is doubled compared to the harvester without magnetic confinement boundary. The magnetic pole spacing of the boundary can be flexibly adjusted according to the ambient wind speed to produce a sufficiently high output power.

## Figures and Tables

**Table 1:** The ratio of the average peak voltage output of wind energy harvesters with magnetic constraints (d=21 mm) to that of the harvesters without magnetic boundaries (wind speed range: 0 to 5.5 m/s).

Wind speed (m/s)	1.5	2	2.5	3	3.5	4	4.5	5	5.5
Voltage ratio	0.87	0.83	0.70	0.72	0.76	0.71	0.70	0.70	0.75

**Table 2:** Fitting coefficients for measuring magnetic force.

**Coefficients**	$a_{0}$	$a_{1}$	$a_{2}$	$a_{3}$	$a_{4}$
Values	5.151	$-2.156\times 10^{3}$	$4.790\times 10^{5}$	$-6.37\times 10^{7}$	$5.253\times 10^{9}$
**Coefficients**	$a_{5}$	$a_{6}$	$a_{7}$	$a_{8}$	$a_{9}$
Values	$-2.732\times 10^{11}$	$8.959\times 10^{12}$	$-1.792\times 10^{14}$	$1.995\times 10^{3}$	$-9.472\times 10^{15}$

## Data Availability

The original contributions presented in the study are included in the article, further inquiries can be directed to the corresponding author.
